# Regional Increases in Incidence of Coccidioidomycosis (Valley Fever) — Arizona, 2005–2022

**DOI:** 10.15585/mmwr.mm7506a3

**Published:** 2026-02-19

**Authors:** Sophia E. Kruger, Irene Ruberto, Thomas Williamson, Justin V. Remais, Alexandra K. Heaney, Jennifer R. Head

**Affiliations:** ^1^University of Michigan, School of Public Health, Ann Arbor, Michigan; ^2^Bureau of Infectious Disease Services, Arizona Department of Health Services; ^3^School of Public Health, University of California Berkeley, Berkeley, California; ^4^The Herbert Wertheim School of Public Health and Human Longevity Science, University of California San Diego, San Diego, California; ^5^Institute for Global Change Biology, University of Michigan, Ann Arbor, Michigan.

**Summary**

**What is already known about this topic?**

Approximately 95% of U.S. coccidioidomycosis (Valley fever) cases are reported from Arizona and California. Incidence of coccidioidomycosis in Arizona approximately doubled during 2005–2022, with most cases in the southwestern counties of Maricopa, Pima, and Pinal.

**What is added by this report?**

A regional analysis of 2005–2022 Arizona coccidioidomycosis incidence data found that although the majority (95%) of cases are reported in the southwestern Sonoran Desert region, the largest relative increases in incidence occurred in the low-incidence northern Plateaus and Mojave Desert regions. Causes of this relative increase are likely multifactorial.

**What are the implications for public health practice?**

Coccidioidomycosis diagnoses are increasing in historically low-incidence regions of Arizona. Directing resources and outreach campaigns to these areas, as well as to regions with historically higher incidence, might increase awareness and guide prevention strategies in low-incidence regions. Ongoing study of the causes of this changing epidemiology could guide tailored regional interventions.

## Abstract

Incidence of coccidioidomycosis (Valley fever), a fungal infection caused by inhalation of *Coccidioides* species spores, has increased substantially across the southwestern United States in association with increasing aridity, warming temperatures, and precipitation volatility. Arizona and California report >95% of U.S. coccidioidomycosis cases, and incidence in Arizona has increased statewide. Patterns within Arizona’s distinct climatological regions have not been characterized, especially in regions outside the known zone of persistently high levels of disease occurrence (hyperendemicity) in the southwest Sonoran Desert region. In this study, surveillance data reported to the Arizona Department of Health Services since 2005 were used to calculate coccidioidomycosis incidence within six ecological regions. During 2005–2022, annual incidence approximately doubled in Arizona, with >95% of cases reported from the Sonoran Desert region. Although the Plateaus and Mojave Desert regions (in the northern parts of the state) reported <1.5% of Arizona cases during this period, these regions experienced the highest relative increases in incidence from the 2005–2007 period to the 2020–2022 period. During 2020–2022, coccidioidomycosis incidence in the Plateaus region was 6.61 times the incidence during 2005–2007 (95% CI = 4.22–10.30), and in the Mojave Desert region, incidence was 4.50 times that during 2005–2007 (95% CI = 3.45–5.89). The Plateau and Mojave regions also reported the highest relative increases in incidence from the 2014–2016 period to the 2020–2022 period. Large relative incidence increases in northern regions, including cooler and wetter regions generally considered less suitable for *Coccidioides* species establishment and transmission, necessitate targeted public health messaging in these areas and support ongoing investigation into the causes of these increases.

## Introduction

Coccidioidomycosis, or Valley fever, is a fungal disease caused by inhalation of spores of the soil-dwelling *Coccidioides* species (*1*). Although coccidioidomycosis typically results in a self-limited pneumonia-like respiratory illness, approximately 5% of patients develop disseminated disease with chronic sequelae, and <1% experience severe pulmonary complications (*1*). Treatment of Valley fever depends on infection severity, disease presentation, and patient immune status and comorbidities, but can involve short-term (3–6 months) oral azole therapy (most often fluconazole or itraconazole) with disseminated or chronic infections often requiring longer treatment (≥1 year to lifelong) (*1*). Coccidioidomycosis is endemic to the southwestern United States, with Arizona and California reporting >95% of U.S. cases (*2*). Since 2000, coccidioidomycosis incidence has increased substantially across the southwestern United States in association with increasing aridity, warming temperatures, and precipitation volatility (i.e., sudden, large, or frequent shifts between extremely wet and dry conditions) (*3*–*6*). Arizona temperatures typically peak in July, and precipitation peaks both during the monsoon season (July–September) and winter months (December–March). Coccidioidomycosis incidence is seasonal; the peak in Arizona typically occurs during October–January, which overlaps the second annual rainy season. The increasing incidence in Arizona is predominantly in the hot and arid Sonoran Desert region that includes the counties of Maricopa, Pima, and Pinal, where the disease is hyperendemic (*4*,*7*). However, changes in incidence patterns within Arizona’s various climatological and ecological regions, especially those outside the Sonoran Desert, have not been investigated. This report describes regional trends in coccidioidomycosis incidence in Arizona during 2005–2022, as well as the climatological profiles of regions with the largest relative increases in incidence.

## Methods

### Data Source

Arizona health jurisdictions are required to report coccidioidomycosis cases to the Arizona Department of Health Services (ADHS) using the laboratory component of the Council of State and Territorial Epidemiologists’ case definition. Information on all laboratory-confirmed coccidioidomycosis cases reported to ADHS during 2005–2022, including week of diagnosis* and U.S. Census Bureau tract of patient residence, was obtained. Cases were assigned to one of six modified Environmental Protection Agency level III ecoregions (regions)^†^ by tract. Cases in persons without residence information (including in persons from tribal lands) were excluded from the analysis.

### Estimates of Population, Temperature, and Precipitation

Annual populations for each region were estimated by summing annual population estimates for U.S. Census Bureau tracts whose geometric centers were contained in the region.^§^ Monthly mean temperature and total precipitation within approximately 6.2 miles^2^ (16 km^2^) grid cells were obtained from the PRISM Climate Group. These values were then averaged within the boundaries of each region.

### Analysis of Change in Incidence

Statewide and regional incidences (number of cases per 100,000 population) were calculated. To calculate changes in incidence over time and by region, a single Poisson model was fit to regional annual incident cases with an offset on the log of the annual regional population and fixed effects on region, a six-level categorical measure of 3-year periods to increase stability of the calculated incidences (2005–2007, 2008–2010, 2011–2013, 2014–2016, 2017–2019, and 2020–2022), and the interaction between the 3-year period and region.^¶^ The offset term in the model accounts for differences in population size by region and over time and allows the model to estimate and compare incidences (number of cases per population unit), rather than raw case counts.

In 2009, one of Arizona’s major commercial laboratories changed its coccidioidomycosis diagnostic and reporting practices and began to report all positive enzyme immunoassay (EIA) results as cases, regardless of results from more specific tests (e.g., immunodiffusion). Because of the increased potential for false-positive results, this practice change is believed to have led, in part, to increases in cases reported from 2009 to 2012. Changes to this laboratory’s EIA testing platform in late 2012 align with a decrease in cases through 2013. Whereas the fitted model was used to calculate incidence rate ratios (IRRs) comparing region-specific incidences in all 3-year periods with incidence during 2020–2022, these reporting changes might have affected the reliability and comparability of incidence estimates during the 2008–2010 and 2011–2013 periods. Therefore, this report highlights comparisons of the incidence in the beginning (2005–2007) and midpoint (2014–2016**) of the study period with the incidence at the end of the study period (2020–2022); the periods 2005–2007 and 2014–2016 were both unaffected by diagnostic and reporting changes.

Analyses were performed using R statistical software (version 4.2.3; R Core Team 2023). Because this study constitutes a public health surveillance activity, the work described did not constitute human research and did not require institutional review board review or exemption according to the Common Rule. This study received ADHS approval to conduct research using case surveillance data collected by ADHS.

## Results

### Arizona Regional Coccidioidomycosis Incidence, 2005–2022

**FIGURE 1:**
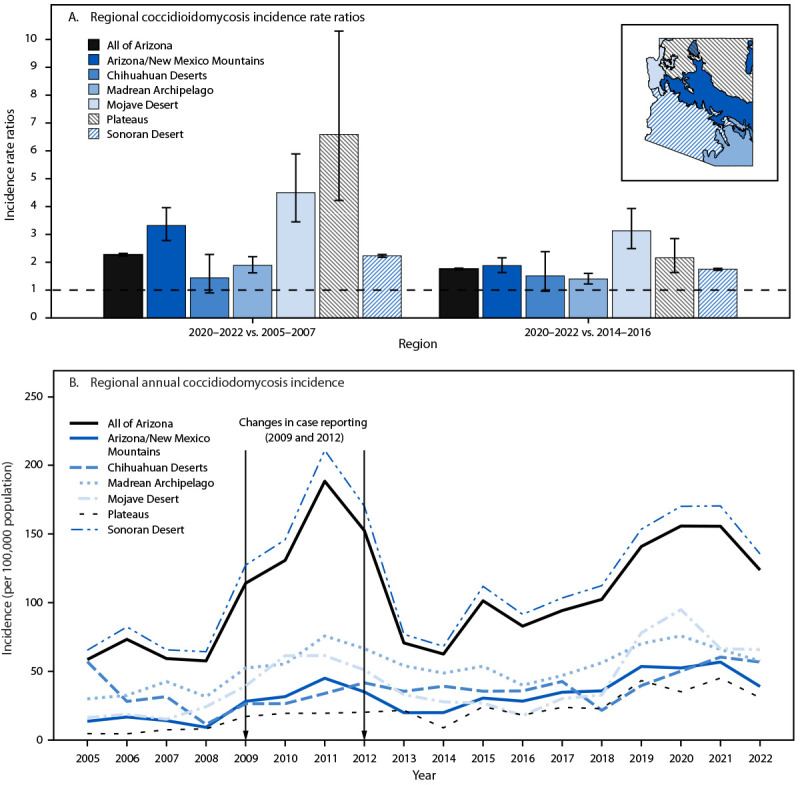
Regional coccidioidomycosis incidence rate ratios* for 2020–2022 versus 2005–2007 and 2014–2016 (A) and annual coccidioidomycosis incidence, by year and region (B) — Arizona, 2005–2022^†^ * With 95% CIs indicated by error bars. ^†^ One study suggests the number of cases reported during 2009–2012 should be decreased by half to adjust for increases attributed to testing changes: Comrie A. No consistent link between dust storms and Valley fever (coccidioidomycosis). GeoHealth. 2021;5:e2021GH000504. https://doi.org/10.1029/2021GH000504

**FIGURE 2:**
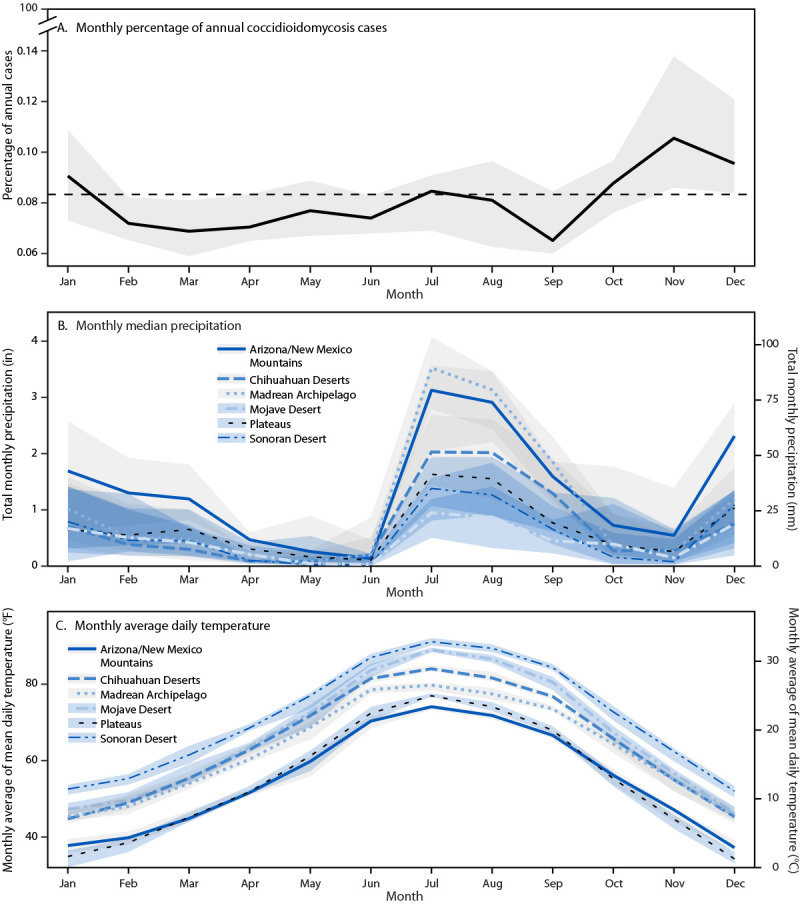
Monthly statewide percentage of annual coccidioidomycosis cases (A),* regional monthly median precipitation (B), and regional monthly average daily temperature (C) — Arizona, 2005–2022^†^ * Dashed line indicates the estimated percentage of cases by month if the disease were not seasonal. ^†^ With IQRs indicated by shaded areas.

During January 1, 2005–December 31, 2022, a total of 152,446 coccidioidomycosis cases were reported in Arizona; among these, 126,982 (83.3%) cases with geographic residence information were included in this study (Table). Median annual incidence was highest (112.13 per 100,000) in the most populous region studied (the southwestern Sonoran Desert region), which reported >95% of cases, and was lowest (19.91 per 100,000) in the northern Plateaus region, which reported <0.5% of cases (Supplementary Figure 1). During this period, statewide annual coccidioidomycosis incidence in Arizona approximately doubled; the average annual 2020–2022 rate (145.02 per 100,000) was 2.27 times that during 2005–2007 (63.73 per 100,000) and 1.76 times that during 2014–2016 (82.28 per 100,000) (Figure 1) (Table). Statewide, the incidence was lowest in 2005 (58.59 cases per 100,000 population) and increased approximately 200% to 188.33 cases per 100,000 in 2011 (Figure 1). After 2011, the incidence decreased through 2014 (62.61 cases per 100,000) and followed a generally increasing trend thereafter, reaching 123.76 cases per 100,000 population in 2022. On average, across the study period, the percentage of reported annual coccidioidomycosis cases peaked in November (Figure 2).

**TABLE:** Number and median annual incidence of coccidioidomycosis cases* and comparison of 2020–2022 with previous 3-year periods, by region (N = 126,982) — Arizona, 2005–2022

Region^¶^	2005–2022		Average annual incidence^†^ of 3-year periods and IRR (95% CI)^§^ comparing incidence with 2020–2022											
			2005–2007		2008–2010		2011–2013		2014–2016		2017–2019		2020–2022	
	No. (column %) of cases	Median annual incidence	No. of cases	IRR (95% CI)	No. of cases	IRR (95% CI)	No. of cases	IRR (95% CI)	No. of cases	IRR (95% CI)	No. of cases	IRR (95% CI)	No. of cases	IRR
Arizona	126,982 (100.00)	101.86	63.73	2.27 (2.22–2.32)	100.84	1.44 (1.41–1.46)	137.16	1.06 (1.04–1.08)	82.28	1.76 (1.73–1.79)	112.49	1.29 (1.26–1.31)	145.02	1 (Ref)
Arizona/New Mexico Mountains	2,074 (1.63)	31.09	14.88	3.32 (2.78–3.96)	23.05	2.14 (1.84–2.49)	33.34	1.48 (1.30–1.69)	26.29	1.88 (1.63–2.16)	41.40	1.19 (1.05–1.35)	49.42	1 (Ref)
Chihuahuan Deserts	183 (0.14)	35.66	38.95	1.44 (0.90–2.28)	21.48	2.59 (1.49–4.52)	37.04	1.51 (0.95–2.38)	36.84	1.51 (0.96–2.38)	34.65	1.61 (1.01–2.55)	55.77	1 (Ref)
Madrean Archipelago	2,364 (1.86)	53.91	35.04	1.90 (1.62–2.20)	46.47	1.43 (1.25–1.64)	65.48	1.01 (0.90–1.15)	47.48	1.40 (1.22–1.60)	57.81	1.15 (1.01–1.31)	66.46	1 (Ref)
Mojave Desert	1,039 (0.82)	32.89	16.80	4.50 (3.45–5.89)	41.68	1.81 (1.50–2.19)	48.53	1.56 (1.31–1.86)	24.18	3.13 (2.49–3.93)	46.99	1.60 (1.34–1.91)	75.76	1 (Ref)
Plateaus	520 (0.41)	19.91	5.60	6.61 (4.22–10.30)	14.96	2.48 (1.84–3.33)	20.50	1.81 (1.38–2.36)	17.15	2.16 (1.63–2.85)	29.99	1.23 (0.98–1.56)	37.04	1 (Ref)
Sonoran Desert	120,802 (95.13)	112.13	71.15	2.23 (2.18–2.28)	112.48	1.41 (1.38–1.43)	152.69	1.04 (1.02–1.06)	90.53	1.75 (1.71–1.78)	123.07	1.28 (1.26–1.31)	158.63	1 (Ref)

**Abbreviations:** IRR = incidence rate ratio; Ref = referent.

* Total cases exclude cases in tribal lands and others that could not be linked to a residence.

^✝^ Number of cases per 100,000 population.

^§^ *Arizona/New Mexico Mountains:* parts of Apache, Coconino, Gila, Greenlee, Mohave, Navajo, and Pinal counties; *Plateaus:* Apache, Coconino, and Navajo counties and parts of Mohave and Yavapai counties; *Chihuahuan Deserts:* parts of Greenlee and Graham counties; *Madrean Archipelago:* Cochise and Santa Cruz counties and parts of Graham, Pima, and Pinal counties; *Mojave Desert:* parts of Mohave county; and *Sonoran Desert:* Gila, La Paz, Maricopa, Yavapai, and Yuma counties and parts of Mohave, Pima, and Pinal counties.

^¶^ IRRs and 95% CIs were calculated using a Poisson model fit to annual regional case data and compared incidence rates during 2020–2022 with incidence rates in each previous 3-year period.

### State and Regional Climate Profiles

During the study period, the Sonoran Desert region experienced the highest temperatures (average annual daily temperature range = 69.71°F –72.43°F [20.95°C –22.46°C]) (Supplementary Table). The more northern Mojave Desert was the driest region (total annual precipitation = 3.84–11.39 in [97.65–289.32 mm]), whereas the southeastern Chihuahuan Deserts and nearby Madrean Archipelago reported more total annual precipitation on average than did the Mojave and Sonoran Desert regions (5.35–13.86 in [135.77–352.22 mm] and 8.27–18.44 in [210.13–468.48 mm]) (Figure 2). The Plateaus region of northeastern Arizona was approximately 44.6°F (7°C) cooler than all desert regions and received on average 2.95 in (75 mm) more precipitation annually than did the Sonoran and Mojave Desert regions. Although temperatures in the midland Arizona/New Mexico Mountains and the Plateaus regions were similarly cool, the midland Arizona/New Mexico Mountains region was the wettest region during the study period (maximum annual precipitation = 23.60 in [599.53 mm]).

### Changes in Regional Coccidioidomycosis Incidence

Coccidioidomycosis incidence in Arizona followed a generally increasing trend across the study period (Supplementary Figure 2). When comparing regional incidences from 2005–2007 to those during 2020–2022, the highest relative increases were reported by the two northernmost regions (the Plateaus and the Mojave Desert regions). During 2020–2022, coccidioidomycosis incidence in the Plateaus region was 6.61 times the incidence during 2005–2007 (95% CI = 4.22–10.30), and in the Mojave Desert region, incidence was 4.50 times that during 2005–2007 (95% CI = 3.45–5.89), corresponding to increases in incidence of approximately 550% and 350%, respectively (Figure 1) (Table). Comparatively, the Sonoran Desert region, where coccidioidomycosis is hyperendemic, reported a 123% increase in incidence between these periods (IRR = 2.23; 95% CI = 2.18–2.28). This equates to absolute average annual increases of 31.44, 58.96, and 87.48 cases per 100,000 population from 2005–2007 to 2020–2022 for the Plateaus, Mojave Desert, and Sonoran Desert regions, respectively. The incidence in all other regions increased from 2005–2007 to 2020–2022.

The incidence also increased between 2014–2016 and 2020–2022, with all regions reporting IRRs from 1.40 to 3.13 for this period (Figure 1). The highest IRR during this more recent period was observed in the Mojave Desert region (IRR = 3.13; 95% CI = 2.49–3.93), followed by the Plateaus region (IRR = 2.16; 95% CI = 1.63–2.85). In absolute terms, average annual incidences for 2020–2022 were 19.89, 51.58, and 68.10 cases per 100,000 population higher than those during 2014–2016 for the Plateaus, Mojave Desert, and Sonoran Desert regions, respectively.

## Discussion

The hot, dry southwestern Sonoran Desert region where coccidioidomycosis is hyperendemic was the largest contributor to absolute increases in incidence in Arizona during 2005–2022. However, this regional analysis indicates that the northern Plateaus and Mojave Desert regions experienced the largest relative increases in incidence compared with other regions during the same period. From 2005–2007 to 2020–2022, incidence increased approximately 550% and 350% in the Plateaus and Mojave Desert regions, respectively, compared with a 123% increase for the Sonoran Desert region.

Possible determinants of the sharp increases in incidence during the study period in more northern parts of the state include 1) changes to regional population susceptibility, in part affected by increased migration to the state, which increased most among older populations during the study period. Older populations are more likely to have symptomatic infection, seek care, and receive a diagnosis; 2) increased disease awareness and reporting; and 3) increased travel (e.g., for work, recreation, leisure, or temporary relocation) from areas of lower to higher endemicity. A 2022 study found clinical *Coccidioides* isolates from patients in northern Arizona clustered with isolates from patients in Maricopa and Pima counties, suggesting that some cases in the northern regions are associated with travel to areas of higher endemicity (*8*). Geographic expansion of *Coccidioides* within historically cooler climates is also a potential cause and is in part supported by a regional analysis in California that documented disproportionate increases in coccidioidomycosis incidence within cooler, wetter regions that were outside the hyperendemic hot and dry Southern San Joaquin Valley (*9*), similar to the findings in this study. Additional research is needed to explain the relative contribution of regional climate and other environmental, socioeconomic, and behavioral contributors to the observed increases and shifts in incidence.

### Limitations

The findings in this report are subject to at least four limitations. First, incidences are underestimates because of missed diagnoses, the low percentage of patients who seek care for mild illnesses, and exclusion of cases identified in tribal lands (*10*). At the same time, the changes in diagnostic testing and reporting practices that occurred in 2009 and 2012 might have led to overestimated case counts during 2009–2013. Accordingly, increases in incidence might be attributed, in part, to increased awareness of coccidioidomycosis testing and case-reporting practices, especially in regions with lower levels of endemicity. To address this possibility, the comparative analyses used in this study focus on the more reliable beginning and midpoint periods as reference periods. Second, areas with low case counts also have smaller populations, which might lead to unstable calculated incidences. This analysis attempted to minimize such instability by using 3-year periods in calculating incidence. Third, because cases are assigned to the region of residence rather than the region of exposure, this analysis was unable to differentiate locally acquired cases from travel-associated cases. Finally, because the surveillance database lacked information on patient ages, incidences were not age-adjusted; however, with minor deviations, population age distributions among adults were similar across regions.

### Implications for Public Health Practice

This regional analysis of Arizona coccidioidomycosis surveillance data highlights disease trends and areas where incidence is increasing most rapidly. Although most cases continue to be reported from a single highly populated zone of hyperendemic disease, including the typically low-incidence regions of Arizona in public health messaging campaigns is important for increasing public and provider awareness about the disease. Although primary prevention of coccidioidomycosis is challenged by the lack of a vaccine and limited understanding of the geographic distribution of *Coccidioides* spp., increasing awareness of coccidioidomycosis can prompt earlier diagnosis and treatment and might motivate adoption of dust control measures (e.g., via vegetation planting, selective irrigation, or both) within towns, work sites, and residences as well as use of personal protective equipment (e.g., N-95 masks) in especially high-risk scenarios, where feasible (*2*).

## Footnotes

* Based on the earliest date among the following: symptom onset, specimen collected for laboratory testing, laboratory test results finalized for a specimen, diagnosis, case reported to the county, entered into the Medical Electronic Disease Surveillance Intelligence System (MEDSIS), reported to ADHS, or submitted to the state. Event date definitions are outlined in Arizona Department of Health Services’ User Training Guide | MEDSIS.

† The EPA level III ecoregions in Arizona include the Arizona/New Mexico Mountains, Arizona/New Mexico Plateau, Chihuahuan Deserts, Colorado Plateaus, Madrean Archipelago, Mojave Basin and Range (Mojave Desert), and the Sonoran Basin and Range (Sonoran Desert). For this analysis, Arizona/New Mexico Plateau and Colorado Plateaus were grouped into a single Plateaus region given ecological similarity and spatial proximity. Shapefiles for these regions were joined in QGIS, a spatial visualization and decision-making tool, to summarize incidence and climate variables for the aggregated region.

§ Annual population per U.S. Census Bureau tract were calculated using tract-level data from the Decennial U.S. Census Demographic and Housing Characteristics File and county-level data from the Arizona Office of Economic Opportunity. The proportion of 2020 U.S. Census Bureau tract populations to county population proportions were calculated and multiplied by annual county population estimates for each year in the study period to obtain tract estimates for all years.

¶ A six-level categorical variable that describes the 3-year period (e.g., 2005–2007) to which each year belongs was included in the model. The model automatically treated one of these 3-year periods as the reference and estimates coefficients on the remaining five of the six 3-year periods. In this way, five fixed effects on the 3-year periods were included in the model.

** The 2014–2016 period included the true midpoint (2014) for the 2005–2022 study period. The 2011–2013 period was excluded as a main period of comparison because case counts in all years during this period were unreliable owing to changes in diagnostic and reporting practices.
